# Trends in Household Salt Iodization and Program Monitoring under the National Iodine Deficiency Disorders Control Programme in Birbhum, West Bengal, India: 2020-2022

**DOI:** 10.7759/cureus.116520

**Published:** 2026-09-18

**Authors:** Manoj Ghosh, Jayita Pal, Priya Debnath, Tanudipta Halder, Anil K Mandi, Paramesh Das, Dinesh Rawat, Yugojyoti Mukherjee, Neloy Chatterjee, Jayanta Nandi

**Affiliations:** 1 Public Health, Institute of Public Health, Kalyani, IND; 2 Epidemiology, All India Institute of Hygiene and Public Health, Kolkata, IND; 3 Community Medicine, L. R. Shah Homoeopathy College, Rajkot, IND; 4 Research Methodology and Biostatistics, L. R. Shah Homoeopathy College, Rajkot, IND; 5 Organon of Medicine, L. R. Shah Homoeopathy College, Rajkot, IND; 6 Pathology and Microbiology, L. R. Shah Homoeopathy College, Rajkot, IND; 7 Physiology and Biochemistry, L. R. Shah Homoeopathy College, Rajkot, IND; 8 Materia Medica, Birbhum Vivekananda Homoeopathic Medical College and Hospital, Birbhum, IND; 9 Practice of Medicine, L. R. Shah Homoeopathy College, Rajkot, IND

**Keywords:** accredited social health activist, household surveillance, iodine adequacy, programme performance, routine monitoring

## Abstract

Background

Universal salt iodization is the cornerstone strategy for the prevention and control of iodine deficiency disorders. This study assessed trends in the household availability of adequately iodized salt and evaluated program monitoring performance under the National Iodine Deficiency Disorders Control Program (NIDDCP) in Birbhum district, West Bengal, India.

Methodology

We conducted a retrospective analysis of routine NIDDCP monitoring data using monthly reports from the District Integrated Disease Surveillance Programme (IDSP) Cell, Birbhum Health District. Data for the period from January 2020 to September 2022 were provided by the Birbhum Health District IDSP Cell with permission for analysis and were analyzed accordingly. Indicators assessed included ASHA participation, household salt sample collection, achievement of monitoring targets, the proportion of tested salt samples with an iodine concentration ≥15 ppm, and block-wise program performance. Descriptive analysis was performed using Microsoft Excel 2019.

Results

The number of Accredited Social Health Activists (ASHAs) engaged in household salt sample collection increased from 1,514 in 2020 to 1,676 in 2021 and 1,724 in 2022. Program performance varied across blocks, with some areas consistently achieving monitoring targets while others showed interruptions and fluctuations. Iodine adequacy among tested salt samples remained high across the district, with Suri I, Suri II, Khoyrasole, and Suri Urban Local Body achieving 100% adequacy. At the district level, 623,532 (95.38%) of 653,765 tested household salt samples had an iodine concentration ≥15 ppm during January-September 2022. Routine salt sample collection was interrupted in several blocks during 2020 and early 2021, with collection resuming during the later observation period.

Conclusions

Routine NIDDCP monitoring in Birbhum district demonstrated sustained high iodine adequacy in tested household salt samples despite operational disruptions during the COVID-19 pandemic. Strengthening routine surveillance systems, ensuring continuity of community-based monitoring, and utilizing program-generated data for decision-making are essential to sustain progress toward elimination of iodine deficiency disorders.

## Introduction

Iodine is an essential trace element required for the synthesis of thyroid hormones thyroxine (T4) and triiodothyronine (T3), which regulate growth, neurological development, metabolism, and reproductive functions across the life course [1,2]. Inadequate iodine intake results in a spectrum of iodine deficiency disorders (IDDs), including goiter, hypothyroidism, impaired cognitive and psychomotor development, adverse pregnancy outcomes, and reduced physical and intellectual productivity [1-3]. Prevention and control of iodine deficiency, therefore, remain important public health priorities globally [4].

Universal salt iodization (USI) is recognized as the most effective, safe, and sustainable population-based strategy for preventing iodine deficiency [1-3]. The World Health Organization (WHO) recommends that household salt should contain ≥15 parts per million (ppm) iodine to ensure adequate iodine intake under normal dietary conditions [5]. Regular monitoring of salt iodine concentration is an essential component of IDD control programs, allowing assessment of iodized salt quality and ensuring that adequately iodized salt reaches households [5].

Implementation of USI has resulted in substantial global progress in reducing iodine deficiency; however, maintaining these achievements requires continuous surveillance and quality assurance mechanisms. The Iodine Global Network has emphasized that sustained monitoring of iodine nutrition and program performance remains essential to identify gaps and prevent reversal of progress, particularly in settings where program implementation varies over time [1].

In India, the National Goitre Control Programme (NGCP) was initiated in 1962 and subsequently expanded and renamed the National Iodine Deficiency Disorders Control Programme (NIDDCP) in 1992 to address the wider spectrum of IDDs. The program aims to reduce the burden of IDDs through universal access to adequately iodized salt, periodic surveys, monitoring of salt iodine content and urinary iodine excretion, quality assurance of iodized salt, health education, and routine program surveillance [6,7]. Under NIDDCP, community-based monitoring activities, including household salt sample collection and testing using salt testing kits, generate routine program data for assessing implementation performance and iodine adequacy among sampled households [6].

Based on iodine concentration, household salt samples were classified as adequately iodized when the iodine concentration was ≥15 ppm and inadequately iodized when the concentration was <15 ppm [5-7]. The India Iodine Survey (2018-2019) reported that approximately four out of five households in West Bengal consumed adequately iodized salt, indicating continued progress along with the need for sustained monitoring [8]. Earlier epidemiological investigations identified Birbhum district as an endemic area for iodine deficiency among school children; however, subsequent evidence suggested improvement in iodine status following sustained program implementation [9]. The National Family Health Survey (NFHS-5) reported that 93.3% of households in Birbhum had access to iodized salt, reflecting considerable progress while indicating that universal coverage had not yet been achieved [10].

Although periodic surveys provide important estimates of iodine status and household access to adequately iodized salt, routine program monitoring data can provide complementary information on program implementation, continuity, and operational performance. However, such routinely generated data from the NIDDCP have been less frequently examined longitudinally at the sub-district level, particularly during periods of health system disruption. The period from January 2020 to September 2022 was selected because routine program monitoring data for this period were available from the Birbhum Health District Integrated Disease Surveillance Program Cell and encompassed the period of major health system disruption associated with the COVID-19 pandemic, followed by subsequent resumption of routine program activities. Therefore, this study aimed to assess temporal trends in household salt iodine adequacy among monitored samples and evaluate program monitoring performance under the NIDDCP in Birbhum Health District, West Bengal, using routinely generated district-level monitoring data from January 2020 to September 2022.

## Materials and methods

Study design and setting

A retrospective descriptive longitudinal analysis of routine program monitoring data was conducted under the NIDDCP in Birbhum Health District, West Bengal, India. Birbhum district has historically been recognized as an IDD-endemic area in West Bengal [9]. During the study period, the district comprised 11 rural administrative blocks, namely Suri I, Suri II, Khoyrasole, Dubrajpur, Rajnagar, Mohammad Bazar, Sainthia, Bolpur Sriniketan, Labpur, Illambazar, and Nanoor. Data from Suri Urban Local Body (ULB) and Bolpur ULB, where NIDDCP monitoring activities were initiated during 2022, were included wherever available.

Study period

The study period was January 2020 to September 2022. This period was selected because it provided a continuous sequence of routine program monitoring data covering the period of major disruption to community-based health services during the COVID-19 pandemic and the subsequent restoration of household salt monitoring activities. The analysis therefore aimed to examine program continuity and performance across this period rather than provide a contemporary estimate of iodine status.

Data source

Routine monthly NIDDCP monitoring reports were obtained from the District Integrated Disease Surveillance Programme (IDSP) Cell, Office of the Chief Medical Officer of Health, Birbhum Health District. The reports contained information on household salt sample collection and testing by participating administrative units and Accredited Social Health Activists (ASHAs). Data extracted for analysis included the number of ASHAs engaged in household salt sample collection, monthly sample-collection targets, number of samples collected and tested, iodine concentration category of tested samples, and block-level monitoring status. The monthly records were compiled chronologically for the study period, checked for completeness and consistency, and reviewed for potential duplication before analysis.

Program monitoring and operational definitions

Under the operational framework of the NIDDCP, each ASHA is expected to collect and test 50 household salt samples per month using standardized Salt Testing Kits [6,7]. Based on iodine concentration, samples with iodine concentrations ≥15 ppm were classified as adequately iodized, whereas those with concentrations <15 ppm were classified as inadequately iodized [5,6].

The primary outcome indicator was the proportion of tested household salt samples with an adequate iodine concentration (≥15 ppm) among samples collected through routine NIDDCP monitoring. Program implementation was assessed using operational indicators, including target achievement, the average number of samples collected per ASHA per month, and program interruptions.

Target achievement was calculated as the percentage of household salt samples collected and tested relative to the expected monthly target:

$\text{Target achievement (\%)} = \frac{\text{Number of samples collected and tested}}{\text{Expected number of samples}} \times 100$

The average number of samples collected per ASHA per month was calculated by dividing the total number of household salt samples collected during the month by the number of ASHAs engaged in sample collection during that month:

$\text{Average samples per ASHA per month} = \frac{\text{Total household salt samples collected during the month}} {\text{Number of ASHAs engaged in sample collection during the month}}$ 

A program interruption was defined as any month during which zero household salt samples were collected in a particular administrative block:

$\text{Programme interruption} = \begin{cases} 1, & \text{if household salt samples collected in the block during a month} = 0,\\ 0, & \text{if household salt samples collected in the block during a month} > 0. \end{cases}$

Routine salt sample testing under NIDDCP was interpreted as an indicator of program implementation and iodine adequacy among monitored households and was not considered a population-representative estimate of household iodized salt coverage.

Study variables

Block-level indicators included ASHA participation, achievement of the monthly sample-collection target, the average number of samples collected per ASHA per month, program interruptions, and the proportion of tested samples meeting the iodine adequacy threshold.

Monthly trends in household salt sample collection, program interruptions, and iodine adequacy among tested samples were assessed during the study period. Block-level variations in program implementation, continuity of monitoring activities, and iodine adequacy among sampled households were also examined.

Data analysis

Data were compiled, cleaned, and analyzed using Microsoft Excel 2019. Descriptive statistical methods, including frequencies, percentages, and summary measures, were used to assess program performance.

The percentage achievement of the targeted salt sample collection, the average number of samples collected per ASHA per month, and the proportion of adequately iodized salt samples were calculated at the district and block levels. Trends in program performance over time were presented using tables and graphical representations.

Monthly fluctuations in salt sample collection were analyzed to assess program continuity during the COVID-19 pandemic and the subsequent recovery period. The proportion of adequately iodized salt samples identified through routine program monitoring was descriptively compared with household iodized salt coverage reported for Birbhum district in the National Family Health Survey-5 (NFHS-5) [8]. However, these indicators were interpreted separately because they were derived from different methodologies and served different monitoring purposes. No inferential statistical testing was performed, as the analysis was intended to describe program implementation trends and operational performance.

Ethical considerations

The study was based exclusively on anonymized secondary program monitoring data obtained from the District IDSP Cell, Birbhum Health District, with administrative permission from the Office of the Chief Medical Officer of Health. The dataset contained no individual-level identifiers, and no direct interaction with human participants was involved. Therefore, informed consent was not applicable. Confidentiality of the program data was maintained throughout the study.

## Results

Program implementation and ASHA participation

The number of ASHAs engaged in household salt sample collection under the NIDDCP increased from 1,514 in 2020 to 1,676 in 2021 and 1,724 in 2022, indicating an overall expansion of program implementation across Birbhum district. The increase in 2022 was partly attributable to the inclusion of Suri ULB and Bolpur ULB in routine program monitoring.

Among the rural blocks, Dubrajpur consistently had the highest number of ASHAs involved in household salt sample collection, followed by Illambazar and Labpur, whereas Rajnagar had the smallest ASHA workforce. Khoyrasole had no ASHA participation in household salt sample collection during 2020, with program activities commencing in 2021. The block-wise distribution of ASHAs engaged in NIDDCP monitoring is presented in Table 1.

**Table 1:** Number of ASHAs participating in household salt sample collection under the NIDDCP in Birbhum district, 2020-2022. ULB, Urban Local Body; NIDDCP, National Iodine Deficiency Disorders Control Programme

Sl. no.	Block	2020, *n* (%)	2021, *n* (%)	2022, *n* (%)
1.	Suri I	166 (10.96%)	166 (9.90%)	167 (9.69%)
2.	Suri II	128 (8.45%)	128 (7.64%)	128 (7.42%)
3.	Khoyrasole	-	156 (9.31%)	156 (9.05%)
4.	Dubrajpur	211 (13.94%)	205 (12.23%)	205 (11.89%)
5.	Rajnagar	59 (3.90%)	57 (3.40%)	57 (3.31%)
6.	Mohammad Bazar	122 (8.06%)	122 (7.28%)	128 (7.42%)
7.	Sainthia	148 (9.78%)	148 (8.83%)	148 (8.59%)
8.	Bolpur Sriniketan	144 (9.51%)	144 (8.59%)	143 (8.29%)
9.	Labpur	176 (11.62%)	177 (10.56%)	178 (10.32%)
10.	Illambazar	184 (12.15%)	194 (11.57%)	194 (11.25%)
11.	Nanoor	176 (11.62%)	179 (10.68%)	179 (10.38%)
12.	Suri ULB	-	-	24 (1.39%)
13.	Bolpur ULB	-	-	17 (0.99%)
	Birbhum district	1,514 (100%)	1,676 (100%)	1,724 (100%)

Program monitoring performance and iodine adequacy among monitored samples

Program monitoring performance varied across administrative blocks during the study period. The average number of household salt samples collected per ASHA per month varied in relation to the NIDDCP target of 50 samples per ASHA per month. Suri II and Bolpur Sriniketan consistently achieved the prescribed monthly target, whereas Mohammad Bazar achieved the target during later periods of monitoring. Khoyrasole demonstrated the initiation and strengthening of monitoring activities after program commencement in 2021. Labpur also showed increased sample collection following improvements in program implementation. In contrast, Illambazar showed reduced sample collection intensity during 2022 following repeated interruptions in monitoring activities (Figure 1).

**Figure 1:**
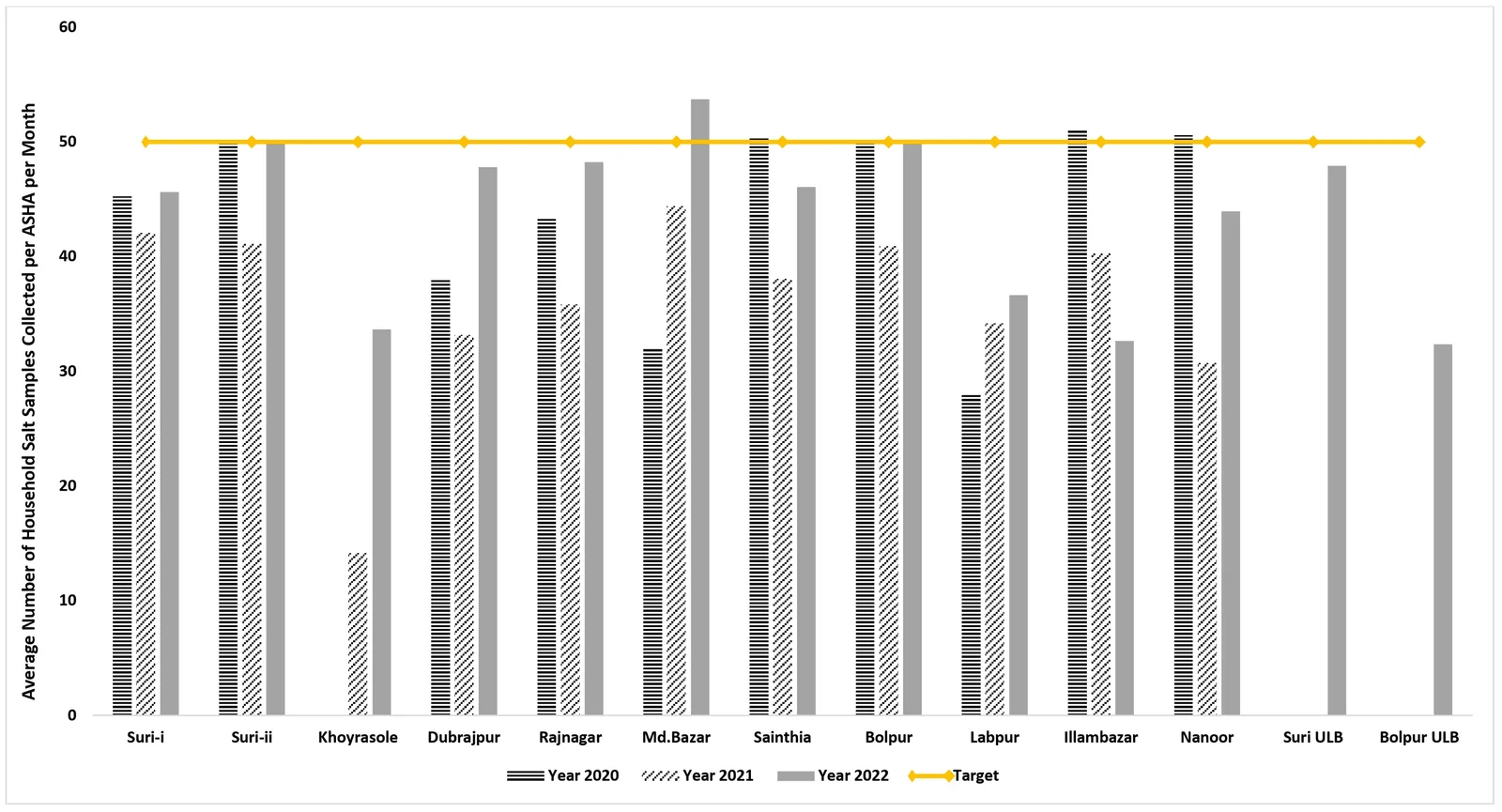
Block-wise average number of household salt samples collected per ASHA per month compared with the NIDDCP target (50 samples/month), Birbhum district, 2020-2022. ULB, Urban Local Body; NIDDCP, National Iodine Deficiency Disorders Control Programme; ASHA, Accredited Social Health Activist

The proportion of tested household salt samples meeting the recommended iodine concentration threshold (≥15 ppm) remained high across the district. Suri I, Suri II, Khoyrasole, and Suri ULB achieved 100% adequacy among tested samples, with all tested samples containing iodine concentrations ≥15 ppm. Other blocks also demonstrated consistently high iodine adequacy, although intermittent reductions were observed in some areas, particularly Mohammad Bazar and Illambazar. At the district level, more than 95% of tested household salt samples collected during 2022 had an adequate iodine concentration (≥15 ppm), indicating the sustained presence of adequately iodized salt among households monitored through routine program activities.

Block-wise program monitoring performance is summarized in Table 2.

**Table 2:** Summary of program monitoring performance across administrative blocks under NIDDCP, January 2020-September 2022. ULB, Urban Local Body; NIDDCP, National Iodine Deficiency Disorders Control Programme; ASHA, Accredited Social Health Activist

Block	ASHA available in 2020	Achieved monthly target during study period	Overall sample collection trend	Adequately iodized salt (≥15 ppm) reached 100%	Remarks
Suri I	Yes	No	Improved	Yes	Monitoring affected during the interruption period
Suri II	Yes	Yes	Stable	Yes	Consistent target achievement
Khoyrasole	No	No	Improved	Yes	Program initiated in 2021
Dubrajpur	Yes	No	Stable	No	Highest ASHA workforce
Rajnagar	Yes	No	Stable	No	Lowest ASHA workforce
Mohammad Bazar	Yes	Yes	Improved	No	Fluctuation in iodine adequacy observed
Sainthia	Yes	Yes (2020)	Stable	No	Intermittent interruption periods
Bolpur Sriniketan	Yes	Yes	Stable	No	Consistent target achievement
Labpur	Yes	No	Improved	No	Strengthening of monitoring activities
Illambazar	Yes	Yes (2020)	Declined	No	Repeated interruptions observed
Nanoor	Yes	Yes (2020)	Improved	No	Higher sample collection volume
Suri ULB	No	NA	Program initiated in 2022	Yes	Recently included
Bolpur ULB	No	NA	Program initiated in September 2022	NA	Monitoring initiated late in the study period

Program continuity and interruptions during the study period

Interruptions in household salt sample collection were observed across multiple administrative blocks during the study period, particularly during 2020 and early 2021. Several blocks experienced months with zero sample collection, reflecting temporary disruptions in routine NIDDCP monitoring activities. Khoyrasole and Illambazar experienced the longest interruptions, with no household salt sample collection reported for eight months each during the study period. Sainthia, Bolpur Sriniketan, and Labpur also experienced multiple months of interruption.

The interruption periods coincided with the COVID-19 pandemic, during which routine health service activities were affected. Despite these disruptions, monitoring activities gradually resumed across most blocks during the later observation period. The inclusion of Suri and Bolpur Urban Local Bodies during 2022 further expanded program monitoring coverage. Block-wise interruption patterns are presented in Table 3.

**Table 3:** Interruptions in household salt sample collection under NIDDCP, January 2020-September 2022. ULB, Urban Local Body; NIDDCP, National Iodine Deficiency Disorders Control Programme

Block	Total months with zero collection	Longest interruption period
Suri I	4	April to July 2020
Suri II	4	February to May 2020
Khoyrasole	8	October to December 2020; June to July 2021
Dubrajpur	5	June to July 2021; September to October 2021
Rajnagar	5	April to May 2020; October to December 2020
Mohammad Bazar	5	March to May 2020
Sainthia	6	October to December 2020; June to July 2021
Bolpur Sriniketan	6	March to May 2020
Labpur	6	March to May 2020
Illambazar	8	Multiple interruption periods
Suri ULB	Program initiated in 2022	Not applicable
Bolpur ULB	Program initiated in 2022	Not applicable

Spatial variation in iodine adequacy among monitored household salt samples

Block-level assessment demonstrated variation in the proportion of tested household salt samples meeting the recommended iodine concentration threshold (≥15 ppm). Suri I, Suri II, Khoyrasole, and Suri ULB recorded 100% adequacy, with all tested salt samples from these areas containing iodine concentrations ≥15 ppm during the monitoring period. This indicates satisfactory iodine content among salt samples tested through routine NIDDCP monitoring in these administrative areas. Several other blocks also showed high levels of iodine adequacy and approached the program target of universal availability of adequately iodized salt.

However, intermittent reductions in iodine adequacy were observed in some blocks, particularly Illambazar and Mohammad Bazar, suggesting variability in maintaining consistent availability of adequately iodized salt among monitored households. At the district level, overall program monitoring activities demonstrated improvement over the study period; however, the proportion of tested samples with an iodine concentration ≥15 ppm was slightly lower in 2022 than in 2021. According to NFHS-5, 93.3% of households in Birbhum district reported access to iodized salt [8]. However, this survey-based estimate and the routine NIDDCP monitoring findings represent different indicators and should be interpreted separately (Figure 2).

**Figure 2:**
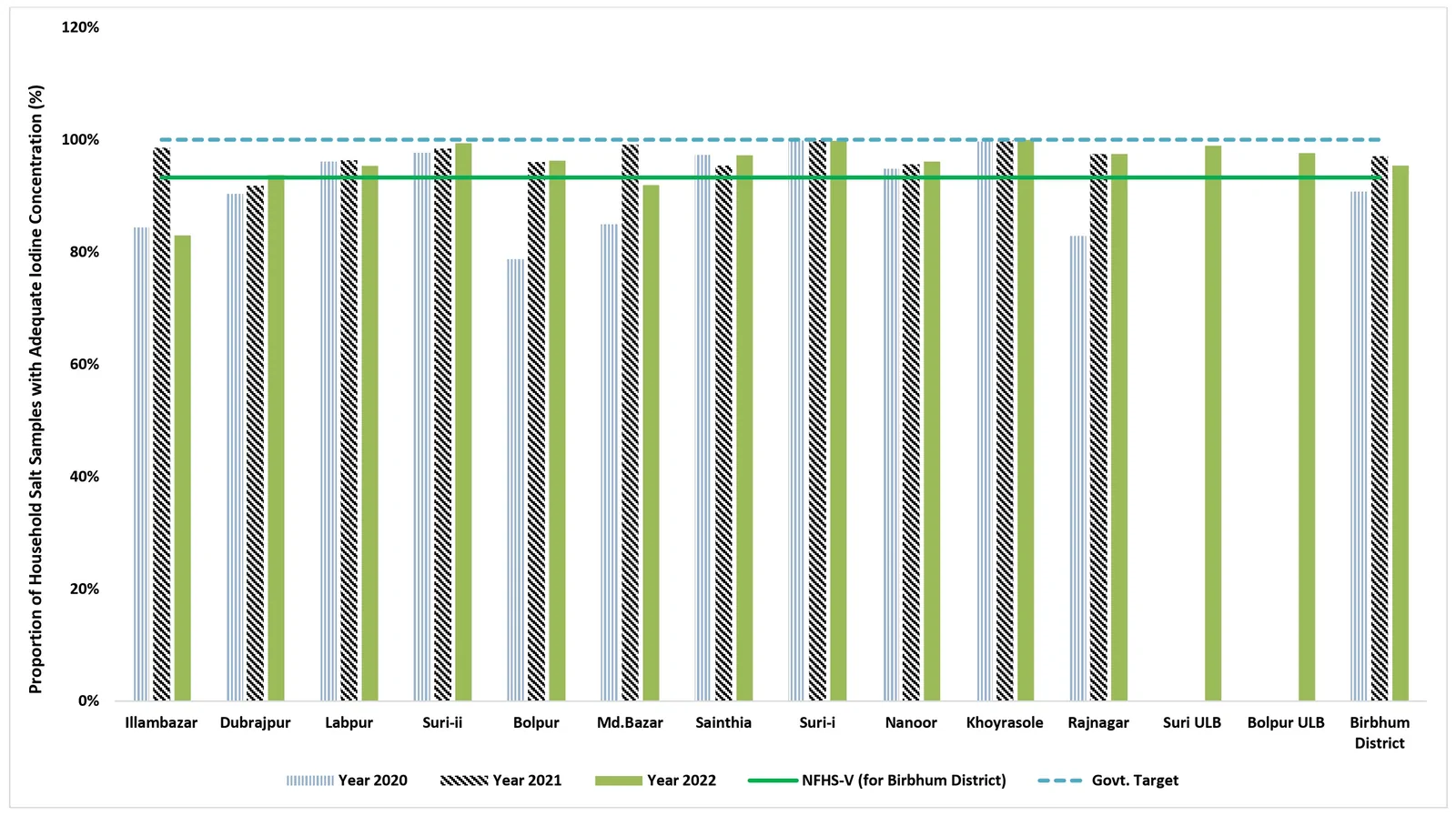
Sub-district-wise proportion of tested household salt samples with adequate iodine concentration (≥15 ppm) under NIDDCP monitoring in Birbhum district, 2020-2022. ULB, Urban Local Body; NIDDCP, National Iodine Deficiency Disorders Control Programme

## Discussion

The present study analyzed routine program monitoring data generated under the NIDDCP in Birbhum district, West Bengal, to assess trends in program implementation, continuity of monitoring activities, and iodine adequacy among household salt samples tested between January 2020 and September 2022. The findings demonstrated expansion of program implementation through increased ASHA involvement, sustained high iodine adequacy among monitored salt samples, and temporary interruptions in routine monitoring activities during the COVID-19 pandemic period. These findings provide important insights into the operational performance and resilience of a long-standing micronutrient intervention program at the district level.

Universal salt iodization has been recognized as one of the most successful public health interventions for the prevention and control of iodine deficiency disorders. Since the introduction of large-scale iodization programs, substantial improvements in iodine nutrition have been achieved globally, with many countries progressing from iodine deficiency toward iodine sufficiency [4,5]. However, maintaining these achievements requires continuous surveillance because iodine status can change over time due to variations in salt production practices, quality control mechanisms, market availability, and consumer behavior [1]. The present study highlights the importance of routine program monitoring systems in identifying operational gaps even in settings where iodine adequacy among monitored samples remains high.

The number of ASHAs engaged in household salt sample collection increased from 1,514 in 2020 to 1,676 in 2021 and 1,724 in 2022, indicating an overall expansion of program implementation capacity in Birbhum district. Community-based monitoring through frontline health workers is a key operational component of NIDDCP, allowing decentralized assessment of iodized salt availability at the household level [6,7]. Integration of surveillance activities within existing primary healthcare systems improves sustainability and enables early identification of implementation challenges [8]. The inclusion of Suri and Bolpur Urban Local Bodies during 2022 further expanded the geographical coverage of routine NIDDCP monitoring.

The study observed high iodine adequacy among tested household salt samples, with several blocks achieving 100% adequacy during the monitoring period. These findings are consistent with the overall improvement in iodine nutrition observed in India following implementation of universal salt iodization. The India Iodine Survey (2018-2019) reported substantial progress in household consumption of adequately iodized salt, although variations across states and districts persist [9]. NFHS-5 also reported that 93.3% of households in Birbhum had access to iodized salt, indicating high coverage but suggesting that universal access has not yet been fully achieved [10]. The present findings complement these survey estimates by demonstrating sustained iodine adequacy among salt samples tested through routine program monitoring.

However, routine monitoring data should be interpreted cautiously, as the present analysis assessed iodine concentration among program-tested samples and does not represent a population-based estimate of household iodized salt coverage. Differences between NFHS-5 estimates and NIDDCP monitoring findings may arise from differences in sampling approach, geographic coverage, frequency of measurement, and purpose of data collection. Routine surveillance systems primarily support program monitoring and early detection of implementation gaps, whereas household surveys provide population-representative estimates; therefore, both approaches should be considered complementary rather than interchangeable [2].

Despite overall satisfactory iodine adequacy, variability in program performance was observed across blocks. Illambazar and Mohammad Bazar demonstrated fluctuations in iodine adequacy and sample collection patterns, suggesting challenges in maintaining consistent program performance. Previous experiences from iodine elimination programs have emphasized that achieving iodine sufficiency does not eliminate the need for continuous quality assurance [1,3]. Sustained district-level monitoring is therefore essential to maintain progress.

An important finding was the disruption of routine salt sample collection during 2020 and early 2021, with the longest interruptions observed in Khoyrasole and Illambazar. These interruptions coincided with the COVID-19 pandemic period, when routine health services, community outreach activities, and field-based surveillance programs were affected globally. Evidence from India and other low- and middle-income countries has shown disruption of essential health services, including maternal and child health services, immunization, disease surveillance, and community-level interventions during the pandemic [11,12]. The gradual restoration of NIDDCP activities in Birbhum demonstrates the resilience of community-based monitoring systems integrated within existing health infrastructure.

The findings emphasize the importance of maintaining surveillance during public health emergencies. Micronutrient programs require continuity even during crises, as interruption of preventive interventions may compromise long-term gains [13]. Strengthening preparedness plans through alternative monitoring mechanisms, digital reporting platforms, and periodic review of program indicators may improve continuity during future disruptions.

The observed decline in the proportion of adequately iodized samples in 2022 compared with 2021, despite overall high adequacy, warrants continued monitoring. Successful iodine deficiency elimination requires sustained political commitment, regulatory enforcement, quality assurance, and regular assessment of household availability [14]. Surveillance systems must continue even after achieving population iodine sufficiency to prevent program fatigue and reversal of progress [15].

The study demonstrates the value of routinely generated program data for health system learning. Such datasets provide information on implementation processes, service continuity, geographic variation, and operational challenges that may not be captured through periodic surveys. Strengthening the use of administrative data can improve program management and accountability [16,17].

Strengths and limitations

The study's strengths include longitudinal analysis of routine program data over nearly three years, including a period of major health system disruption, and assessment of block-level variation in program performance. However, reliance on routine data may have introduced variability in reporting completeness and recording practices. The findings reflect iodine adequacy among monitored household salt samples and are therefore not population-representative estimates. Routine salt testing kit-based assessment was used instead of laboratory-based quantitative estimation, and minor measurement variability cannot be excluded. Although interruptions in sample collection coincided with the COVID-19 pandemic, causality could not be established, and other operational factors such as workforce availability, logistics, supervision, and supply chains were not assessed. Biological indicators of iodine status were also unavailable.

## Conclusions

This study demonstrates that routine monitoring under the NIDDCP in Birbhum district maintained high iodine adequacy among tested household salt samples despite operational disruptions during the COVID-19 pandemic period. The expansion of ASHA involvement, gradual restoration of monitoring activities, and sustained availability of adequately iodized salt indicate the resilience of district-level program implementation. However, variations in monitoring intensity and temporary interruptions across blocks highlight the need for continuous surveillance, supportive supervision, and strengthening of program continuity mechanisms. Systematic analysis of routinely generated program data remains valuable for strengthening surveillance systems, identifying operational gaps, and informing preparedness strategies for future public health emergencies. Sustained investment in monitoring systems, alongside periodic population-based assessments, remains essential to maintain progress toward universal access to adequately iodized salt and the long-term elimination of iodine deficiency disorders.
